# Four anecdotes, four superlative men, and some musings on the meaning of medical physics practice

**DOI:** 10.1002/acm2.13264

**Published:** 2021-05-07

**Authors:** Michael Mills

**Affiliations:** ^1^ Department of Radiation Oncology University of Louisville Louisville KY USA

April 9–10^th^ brought to Louisville, KY a flowering of my dogwood trees and some occasional violets pushing their way through my grass. Spring is a fertile season for new thinking, and I wish to share new ideas. Some discussions I have had with certain medical physicists over the past several days have been especially thought‐provoking and caused me to reflect on some of the character and substance of medical physics practice. I herewith offer some personal anecdotes that resonate with my experience of medical physics education and practice along with some contemporary thoughts.

My first story stems from 1966 and my 8^th^‐grade math class. Dyke Goodin, my instructor, offered that anyone who earned a 100 average on all tests, given weekly, would earn an A+ in the class. I took him up on this challenge as I thought I only had to be careful and I could do it. In 2018, I contacted Dr. Goodin, and asked if he remembered me. I wrote: “One of your challenges to our class, so long ago, was to strive for perfection. Your standard for an A+ was daunting. Nothing less than a 100% average on all weekly tests (with no extra credit) including the final would earn it. I determined I would do it, and indeed, in the final quarter, I earned that elusive and rare A+. Let me tell you what that did for me. I had the confidence I would go as far as I could go in whatever my calling chose for me.” His response: “It might behoove you to know that you were the first student to make a 100 average without bonus points. There have been only 17 young people to accomplish this feat in my 47 years. I remember you very well. You thrived on the challenge of academic excellence. You made a 100 on my most challenging test.” My 50^th^ (plus 1) class reunion is in June, and I hope to see Dr. Goodin in Atlanta for the first time in 54 years. In 1966, the goal to strive for perfection was unquestioned. A career in medical physics may mean over 50,000 calculations, all of them perfect or a patient may suffer injury or worse.

My second anecdote (from 1973) comes from when I was working at Doctor’s Hospital in Tucker, Georgia. A gentleman having a lumbar spine exam was asking a lot of technical questions of our flustered technologist. I was a senior physics major and I volunteered to answer his questions. He asked about the grid, the bucky, the energy, the collimation, the radiation safety procedures in place, and a few other questions; I was able to satisfy him. He asked me what I planned to do, and I told him I was planning to attend the MS program in Health Physics at Georgia Tech next year. He smiled and said he would see me in class. I looked up and remembered his name: Karl Z Morgan. Some of you may recognize who he was: The Director of Health Physics at the Oak Ridge National Laboratories in the 1940s and 1950s, the founder of the Health Physics Society and the first Editor of the Health Physics Journal. He became my advisor for my first Master’s degree.

My third story is from my PhD Advisory Committee meeting at the MD Anderson Cancer Center. My advisor was the renowned Ray Meyn, one of the world’s leading radiobiologists and ultimately the acting Chair of the Department of Experimental Radiotherapy at the MD Anderson Cancer Center. However, in 1977, he was an Assistant Professor just beginning his career and I was his student. I put together a project, which I thought was acceptable. His friend and a member of my committee was a very bright scientist whose name was Roger H_. Every student was afraid of him. After presenting my project, Dr. H_ said, “all of this is good, but what is your goal in accomplishing this project since you want to become a medical physicist?” I replied, “my goal is to do such a good job with the experiments, the writing, the conclusions and the publication of this work that it makes my supervisor, Dr. Meyn, an eminent scholar, known around the world for the value of his creative science.” Ray Meyn chipped in, “Well, now that is a goal and ambition I can wholeheartedly endorse!” Ultimately, the two articles from this project received 156 citations, according to the Web of Science. Ray told me in 2007 that this was the most cited project in his career.

My final anecdote stems from when I was teaching a laboratory in radiation safety in 1979. It was about 7:30PM and the clinic was empty. I had two students, and we had access to the Atomic Energy of Canada Limited (AECL) Therac 20. I set the energy for 18 MV, the jaws for 40 x 40 cm, pointed the unit at 180 degrees (straight up), and told the students to find where the beam was coming out and measure it with the survey meters I provided. After one minute, I activated the beam. After 10 minutes, they returned. The beam was exiting into a physician’s office with the center of the beam right under the chair! The office was occupied by Dr. Bill Spanos, the man who ultimately became Chair of Radiation Oncology at the University of Louisville, and the gentleman who hired me to be Chief of Physics. His message through my students was: “Thank you very much, I appreciate knowing about this.” I later found out that, at least for some of his patients, four field techniques were eliminated in favor of three field wedged techniques, removing the 180‐degree beam.

So many in our culture approach life seeking to obtain power and to put themselves in a position where they do not need to practice excellence and are free from being questioned or criticized about their true goals and ambitions. Today, we can also be faced with significant social criticism of such perfectionistic goals and ambitions in education as a challenge to attain a 100 average. Does our culture welcome critical questions, or are we more often expected to trust powerful authorities without questioning them? Is it all about us and our goals, or is it still about making our employers and supervisors successful? Do we relentlessly strive to eliminate even small risks or do we minimize some significant risks while greatly magnifying others for personal gain or convenience? Is it wise to hold such self‐interested thinking and to welcome and empower those with such attitudes in the medical physics profession? How will this affect the practice of medical physics and ultimately the outcome of our patients?

What things do we not know that are putting ourselves, our patients or our employers at risk? After striving for excellence, asking critical questions, focusing on our goals, and examining the limits of our knowledge in a profession we are spending a lifetime to master, we often still find ourselves quite vulnerable to the unknown. Patients are ultimately best served by medical physicists who strive relentlessly for excellence, in a work environment unafraid to identify deficiencies or errors. Medical physics differs from many contemporary vocations with flexible missions where the time’s social priorities merge naturally with the profession’s flexible mission. At the end of the day, all medical physics work critically and heroically deals with human lives, their conditions improved or harmed by our actions according to the mindset we embrace.

